# Reduction of Cell Surface T-Cell Receptor by Non-Mitogenic CD3 Antibody to Mitigate Murine Lupus

**DOI:** 10.3389/fimmu.2022.855812

**Published:** 2022-03-28

**Authors:** Masashi Morita, Masayuki Mizui, Satoshi Masuyama, George C. Tsokos, Yoshitaka Isaka

**Affiliations:** ^1^ Department of Nephrology, Osaka University Graduate School of Medicine, Suita, Japan; ^2^ Division of Rheumatology, Department of Medicine, Beth Israel Deaconess Medical Center and Harvard Medical School, Boston, MA, United States

**Keywords:** systemic lupus erythematosus, lupus nephritis, autoimmunity, autoantibody, TCR (T-cell receptor), 145-2C11, Fc region, NZB/W F1

## Abstract

T-cells are critically involved in the pathogenesis of systemic lupus erythematosus. Although treatment with the anti-CD3 antibody has been reported to be effective in several autoimmune disease animal models including lupus, the immunosuppressive mechanisms remain obscure because of its pleiotropic *in vivo* kinetics. In this study, a conventional anti-CD3 (2C11C) and a non-mitogenic anti-CD3 with a manipulated Fc region (2C11S) were compared to elucidate the underlying mechanism of action. The efficacy and safety of 2C11S *in vivo* were demonstrated by sustained TCR reduction for a longer period as compared to 2C11C and no induction of cytokine release or T-cell depletion. Anti-CD3s were administered to NZB/W F1 (BWF1) mice at different time points for individual periods. The short-term treatment with 2C11S in the early phase of lupus suppressed the autoantibody associated with the reduction of germinal center B-cells. Treatment in the late phase attenuated lupus nephritis without affecting autoantibodies or differentiation of effector T-cells. The effect of reduced TCR in the development of autoimmunity was examined by CD3ζ heterozygous-deficient mice, in which T-cells had reduced TCR intensity but showed normal TCR signaling response. Autoantibody and lupus nephritis were attenuated significantly in CD3ζ heterozygous-deficient lupus-prone mice. Collectively, the reduction of surface TCR by non-mitogenic anti-CD3 could sufficiently suppress the development of lupus.

## Introduction

Systemic lupus erythematosus (SLE) is an autoimmune disease characterized by autoantibody production and multiple-tissue inflammation and damage. The basis of the pathogenesis in SLE is the breakdown of immune tolerance against autoantigens, and T-cells are important in the development of organ inflammation in SLE. Abnormal signaling and epigenetic changes in T-cells lead to altered gene transcription and cytokine production, which contribute to their aberrant phenotype (1). Therefore, controlling the functional abnormalities in T-cells has a therapeutic potential for lupus.

The anti-human CD3 clone OKT3 was the first T-cell-targeted biological agent found to exert immunosuppressive function and was used to treat allograft rejection. Soon thereafter, OKT3 was withdrawn from the market because of the severe adverse effects including fever and cytokine release syndrome caused by its mitogenicity. However, anti-CD3 has recently been revisited as a tool to treat autoimmune diseases (2). A humanized Fc receptor (FcR) non-binding anti-CD3, teplizumab, has been tested in a clinical setting. It was initially observed to reverse acute renal allograft rejection (3) and was shown to delay the onset of type I diabetes and is currently in a phase III trial (4). Unfortunately, teplizumab was not approved by FDA because of lack of data on its *in vivo* kinetics and/or dynamics rather than because of safety or efficacy concerns (2). Nonetheless, it is still at the forefront of a growing number of trials targeting T-cells to treat autoimmune diseases, while further validations are necessary to examine its mechanism of action.

The features of anti-CD3 have been investigated mainly in murine models. The mouse anti-CD3ε, clone 145-2C11 (2C11), has a similar activity to anti-human CD3 OKT3 (5) and has been shown to be efficacious on various models of autoimmunity and graft-versus-host disease (GVHD) (6–8). Additional studies have suggested the various mechanisms of action responsible for the therapeutic effects: reduction in the density of the T-cell receptor (TCR)/CD3 complex on T-cells (9, 10), T-cell depletion dependent on the functional Fc region and phagocytosis (11), T-cell apoptosis, T-cell anergy (12, 13), and induction of regulatory T-cells (Tregs) (14). However, it is still not clear which mechanism of action is dominant in each model of treatment.

Whether anti-CD3 can suppress lupus remains obscure. Lupus disease severity was reported to be ameliorated following intravenous, oral, or nasal administration of 2C11 F(ab′)2, which was developed to eliminate Fc-mediated mitogenicity (15–17). Various mechanisms of action have been suggested for 2C11: T-cell anergy, T-cell depletion (15), induction of Tregs, and downregulation of effector T-cells (16, 17). However, the F(ab′)2 fragment of anti-CD3 has a very short half-life due to its molecular instability and a sufficiently low molecular weight enough to be filtered through glomerulus (8, 18). Because of its fast clearance, high doses of the F(ab′)2 anti-CD3 were required to exert the expected efficacy. Since the effect of 2C11 F(ab′)2 on lupus has not been compared to that of conventional 2C11 (2C11C), the superiority of F(ab′)2 has not been evaluated. Therefore, the mechanisms of anti-CD3-mediated immunosuppression are still unknown.

In this study, we compared the biological activities of 2C11C and non-mitogenic Fc-manipulated 2C11 (2C11 silent; 2C11S) which loses its ability to bind FcR and complement and examined their effects on disease development in the lupus-prone mice NZB/W F1 (BWF1).

## Material and Methods

### Mice

Female B6 mice at 6–10 weeks of age and female BWF1 mice at 10 or 20 weeks of age were purchased from Japan SLC (Shizuoka, Japan), housed in a specific pathogen-free animal facility at The Osaka University Graduate School of Medicine in accordance with the Animal Committee of Osaka University’s guidelines. B6.MRL-Fas^lpr^/J (B6lpr) and B6.129S4-Cd247^tm1/lov^/J mice were purchased from The Jackson Laboratory (Bar Harbor, ME, USA). B6.129S4-Cd247^tm1/lov^/J mice were backcrossed for at least six generations into C57BL/6J (B6) mice. Mice were bred and housed in a specific pathogen-free animal facility in accordance with the Beth Israel Deaconess Medical Center Institutional Animal Care and Use Committee (IACUC).

### Antibodies and Treatment Procedures

To compare the biological activities of each antibody, female B6 mice at the age of 6–10 weeks received an intraperitoneal injection of 1 mg/kg Armenian hamster 2C11C (ah2C11C; Bio X Cell, Lebanon, NH, USA), mouse 2C11C (m2C11C; Absolute Antibody, Oxford, UK), mouse 2C11S (2C11S; Absolute Antibody), or mouse isotype control immunoglobulin G1 κ (IC; BioLegend, San Diego, CA, USA) 1 or 2 times once a week. To examine the production of the antigen-specific antibody or inflammatory cytokine, B6 mice received 1 mg/kg of each antibody 4 days before (4-hydroxy-3-nitrophenyl) acetyl-chicken gamma globulin (NP-CGG) immunization or TCR restimulation by concanavalin A. Mice immunized with 100 μg of NP-CGG (Ratio 1-9; Wako Pure Chemical Industries, Osaka, Japan) in 100 μl of alum (Wako Pure Chemical Industries) by intraperitoneal injection were sacrificed 7 days after the immunization. To examine temporal T-cell reduction, 25 mg/kg of liposome clodronate or liposome (Hygieia Biosciences, Woodland, CA, USA) was pre-injected 24 h before injection of each antibody, and 5 mg/kg of neutralizing antibodies to integrin LFA-1 and VLA-4 (Bio X Cell) or phosphate-buffered saline (PBS) was pre-injected 3 h before that. To investigate the therapeutic effects of each antibody on lupus, female BWF1 mice at the age of 10 weeks (early phase) or 20 weeks (late phase) received 3 mg/kg of ah2C11C, 2C11S, or IC 4 times once a week as the short-term treatment and were sacrificed at 15 or 25 weeks. In the long-term treatment, they started to receive 3 mg/kg of each antibody once a week from 20 weeks of age. The doses of 2C11S or ah2C11C were adjusted in 3-mg/kg units up to 9 mg/kg while monitoring the TCR density to be 50% or less by flow cytometry. The treatment was continued by 40 weeks of age.

### *In Vitro* Assays

Mouse CD4^+^T-cell, naïve CD4^+^T-cell, and CD3^+^T-cell isolation kits (BioLegend) were used to purify pan CD4^+^T-cells, naive CD4^+^T-cells, and pan T-cells from splenocytes. For the evaluation of pro-inflammatory cytokine production after TCR restimulation, purified splenic pan T-cells were seeded in 48-well plates (Iwaki, Tokyo, Japan) at a density of 5 × 10^5^ cells per well in the presence of 1640 RPMI medium (Gibco, Grand Island, NY, USA) containing 10% inactivated fetal bovine serum (FBS; Biological Industries, Haemek, Israel) and 2 μg/ml concanavalin A (Sigma-Aldrich, St. Louis, MO, USA) for 24 h at 37°C. To examine antibody-induced T-cell phagocytosis by macrophages, pan T-cells labeled with pHrodo Red (Sartorius, Göttingen, Germany) were seeded in 96-well plates (Corning, Tewksbury, MA, USA) at a density of 5 × 10^5^ cells per well and cocultured with bone marrow-derived macrophages (BMDMs) prepared as previously described (19) at a density of 5 × 10^4^ cells in the presence of DMEM high glucose medium (Gibco) containing 5 μg/ml of IC, 2C11S, or ah2C11C, and 10% not inactivated FBS. T-cell phagocytosis by BMDMs was observed for 2 h at 37°C and imaged at 15-min intervals (Sartorius). Thresholds for calling pHrodo Red-positive events were set based on intensity measurements of pHrodo Red-labeled T-cells that lacked BMDMs, and the number of pHrodo red-positive T-cells per field were counted using IncuCyte SX1.

### ELISA and Functional Assays

Interferon (IFN)-γ tumor necrosis factor (TNF)-α, and interleukin (IL)-2 were measured in the supernatant of cell-cultured medium or the plasma using the ELISA system (BioLegend). Anti-mouse dsDNA IgG antibodies in the plasma were measured using the ELISA system (Wako Pure Chemical Industries). For the measurement of plasma NP-specific IgG antibodies, a MaxiSorp 96-well plate (Nunc) was coated with 10 μg/ml of NP-BSA in PBS at 4°C overnight and blocked with 2% normal goat serum in Tris-buffered saline with 0.05% Tween 20 (TBS-T). Plasma samples diluted 1: 100 were incubated at room temperature for 2 h, and subsequently HRP-conjugated goat anti-mouse IgG was incubated for 2 h. HRP activity was evaluated with TMB solution and 1 M sulfuric acid (Sigma Aldrich). The total urinary protein (uTP) and urinary creatinine (uCr) concentrations were measured by clinical diagnostic reagents (Wako Pure Chemical Industries).

### Flow Cytometry and Immunoblot

PBS-perfused kidneys were minced and digested with Liberase TH (Roche) for 30 min at 37°C. Spleens or digested kidneys were passed through a 70-μm cell strainer (Corning). Erythrocytes were eliminated using Red Blood Cell Lysis Buffer (Sigma-Aldrich) for 5 min at room temperature. Isolated cells were stained with the mAbs specific for the following, for 30 min at 4°C: Fc-receptor (93), CD4 (GK1.5), CD8 (53-6.7), CD90.2 (30-H12), TCRβ (H57-597), CD45 (30-F11), CD11b (M1/70), F4/80 (BM8), 7AAD, Zombie Aqua, CXCR5 (L138D7), PD-1 (29F.1A12), B220 (RA3-6B2), GL7 (GL7), CD95 (SA367H8), CD25 (PC61), CD44 (IM7), CD62L (MEL-14), CD73 (TY/11.8), and FR4 (TH6) (all from BioLegend). For intracellular cytokine staining, cells were stimulated with 400 ng/ml PMA (Sigma-Aldrich), 4 μg/ml ionomycin (Sigma-Aldrich), and GolgiPlug (BD Biosciences, Franklin Lakes, NJ, USA) for 4–6 h at 37°C. The mAbs specific for IFN-γ (XMG1.2; BioLegend), IL-17a (TC11-18H10.1; BioLegend), IL-2 (JES6-5H4; BioLegend), FOXP3 (MF-14; BioLegend), and EGR-2 (clone erongr2; eBioscience, San Diego, CA, USA) and the True-Nuclear™ Transcription Factor Buffer Set or Cyto-Fast™ Fix/Perm Buffer Set (BioLegend) were used for detecting intracellular transcription factors or cytokines. Fluo-4 AM (AAT Bioquest, Sunnyvale, CA, USA) was used for the evaluation of intracellular Ca^2+^ influx in T-cells after anti-CD3 antibody or ionomycin loading. T-cells in early apoptosis were stained with the Dual Sensor MitoCasp™ Kit (Cell Technology, Hayward, CA, USA). Quantitative analyses of TCR-Vβ families on thymic CD4 single-positive cells were carried out using the Mouse Vβ TCR Screening Panel Kit (BD Pharmingen, San Diego, CA, USA). Stained cells were evaluated using an LSR II flow cytometer (BD Biosciences) or FACSVerse (BD Biosciences) and analyzed with FlowJo V10 software (Treestar, Woodburn, OR, USA). For immunoblotting, splenic naïve CD4^+^T-cells were stimulated and target proteins were detected with the antibodies specific for phospho-Zap70, phospho-p44/42 MAPK, total p44/42 MAPK, β-actin (All from Cell Signaling Technology), and CD3ζ (Invitrogen) and performed as described previously (20).

### Quantitative Real-Time PCR Analysis

Total mRNA from the homogenized whole kidney by a polytron was purified with TRIzol reagent (Invitrogen, Carlsbad, CA, USA) or RNeasy Mini Kit (Qiagen, Hilden, Germany) and reverse transcribed to generate cDNA (Invitrogen). Real-time SYBR Green PCR analyses were performed using QuantStudio 7 (Applied Biosystems, Foster City, CA, USA). The relative gene expression of *Gapdh* was evaluated by the comparative cycle threshold method. The primer sets are shown in **Table S1**.

### Histological Analyses

Kidneys were fixed in 4% paraformaldehyde. Tissue sections were stained with periodic acid-Schiff. Immunohistochemical staining for Mac-2 was performed by the avidin–biotin complicated method using the biotinylated anti-mouse/human Mac-2 antibody (clone M3/38; BioLegend). The sections were visualized by Nikon (Melville, NY, USA). The histopathological glomerular injury was graded in a semiquantitative manner (0–3^+^) (21) and evaluated blindly by two pathologists for at least 20 glomeruli, and the average was calculated.

### Statistical Analyses

Parametric or non-parametric data were expressed as mean ± standard deviation or median with an interquartile range. The unpaired Student *t* or Mann–Whitney test was used for analyzing the differences between two groups. The paired *t* test was used for time-series tests. One-way ANOVA with Dunnett’s multiple-comparison test or Kruskal–Wallis with Dunn’s multiple-comparison test was used for analyzing the differences between three groups or more. Kaplan–Meier curves and the log-rank test were used for proteinuria and survival test. p values < 0.05 were considered statistically significant. Statistical analyses were performed by GraphPad Prism version 7.04 (GraphPad Software, La Jolla, CA, USA).

## Results

### Alteration of T-Cell Function and Kinetics After Anti-CD3 Treatment

**Figure 1:**
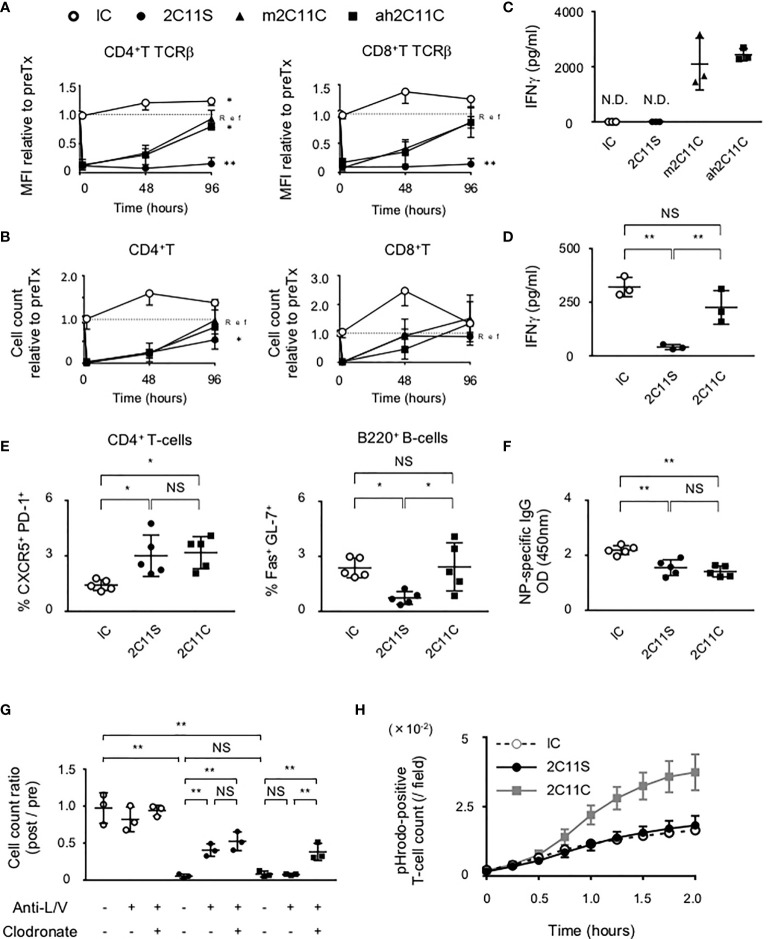
Functional difference between 2C11C and 2C11S. **(A)** TCRβ intensity on CD4^+^ and CD8^+^ T-cells and **(B)** their numbers in peripheral blood was examined. The rate of change in the mean fluorescence intensity and numbers before and after antibody injection was calculated. Time series data in each group; ah2C11C (black triangle), m2C11C (white triangle), 2C11S (square), and IC (circle) were shown. **(C)** Levels of plasma IFN-γ in 4 h after each antibody injection were measured (N.D., not detected). **(D)** IFN-γ production by splenic pan T-cells restimulated with concanavalin A after each antibody treatment was measured. **(E)** Splenic CD4^+^CXCR5^+^PD-1^+^ and B220^+^Fas^+^GL-7^+^ cells after NP-CGG immunization were evaluated in each group. **(F)** Plasma titer of the NP-specific antibody was shown. **(G)** Liposome clodronate and neutralizing antibodies to integrin LFA-1 and VLA-4 (anti-L/V) or PBS were pre-injected before injection of each antibody. Pan T-cell numbers in peripheral blood at 0 and 2 h after each antibody treatment were examined, and the rate of change was calculated (NS, Non Significance, *p < 0.05, **p < 0.01). **(H)** Phagocytosis of pHrodo red-labeled T-cells by BMDMs was counted in time series.

To compare the biological activities of CD3 mAbs, the TCRβ intensity on CD4^+^T and CD8^+^T cells and their numbers in peripheral blood were examined at different time points after a single injection. We noticed a reduction of TCRβ intensity which was maintained 96 h after the injection of non-mitogenic Fc-manipulated 2C11 (2C11 silent; 2C11S), but not after the injection of Armenian hamster conventional 2C11 (ah2C11C) or mouse 2C11C (m2C11C) (**Figure 1A**). The number of CD4^+^T and CD8^+^T-cells was temporally reduced but recovered similarly 96 h after the injection of ah2C11C, m2C11C, and 2C11S (**Figure 1B**). Moreover, ah2C11C and m2C11C, but not 2C11S, induce cytokine release (**Figure 1C**, **S1A**), and the biological activities of ah2C11C and m2C11C are confirmed to be equivalent. We used ah2C11C as a reference for all the following experiments and called it “2C11C.” To investigate the effect of TCR reduction on T-cell functions, sorted splenic pan T-cells after injection of each antibody were restimulated with a lectin, concanavalin A, which binds to TCR and promotes inflammatory cytokine production (22). 2C11S significantly inhibited the secretion of IFN-γ, TNF-α, and IL-2 than mouse isotype control immunoglobulin G1 κ (IC) and 2C11C (**Figure 1D**, **S1B**), indicating that 2C11S suppresses the effector function of T-cells *via* sustained TCR reduction. Next, B6 mice were immunized with a T-cell-dependent antigen, NP-CGG, after each antibody treatment to evaluate NP-specific antibody response. Anti-NP antibody production was reduced with suppressed formation of germinal center B (GCB) cells and NP-specific B-cells in the 2C11S group, whereas Tfh cells were not reduced. In the 2C11C group, anti-NP production and formation of NP-specific B-cells were reduced, while neither Tfh cells nor GCB cells were reduced (**Figures 1E, F**, **S1C**). These results suggested that TCR reduction by 2C11S suppressed GCB formation possibly through a reduced interaction between Tfh and GCB cells, while the 2C11C-mediated reduction of antibody responses was not parallel to the GCB formation.

### Mechanisms of Anti-CD3-Mediated Reduction of T-Cell Number in the Peripheral Blood

The administration of anti-CD3 mAbs has been suggested to activate adhesion molecules in vascular and lymphatic endothelia, ICAM-1 and VCAM-1, as well as those in T-cells, LFA-1, and VLA-4 (23, 24). Here we found that pre-injection of neutralizing antibodies of LFA-1 and VLA-4 partially recovered the number of T-cells in the peripheral blood after 2C11S treatment, but not after 2C11C treatment (**Figure 1G**). This result indicates that 2C11C causes T-cell depletion. Therefore, we checked whether the T-cell reduction was due to apoptosis after 2C11 mAb treatment. Flow cytometry analysis showed that neither 2C11C nor 2C11S induced significant T-cell apoptosis (**Figure S1D**). Next, the effect of T-cell phagocytosis on the reduction of T-cell number was examined by pretreating with liposome clodronate, a marginal zone macrophage depletor (25), in addition to the neutralizing antibodies (**Figure 1G**). Depletion of macrophages by liposome clodronate restored the number of T-cells after 2C11C treatment, indicating that 2C11C induced T-cell phagocytosis by macrophages, which contributes to the reduction of peripheral T-cell number. This was confirmed *in vitro* by the detection system of phagocytosis (**Figure 1H**). Purified pHrodo-labeled T-cells were cocultured with BMDMs in the presence of each antibody. pHrodo-positive T-cell count recorded over time showed that 2C11C but not 2C11S significantly induced T-cell phagocytosis by macrophages (**Figures 1H**, **S1E**). These results indicate that the reduction in the number of T-cells by 2C11S is due to trapping into the vessels through temporal activation of adhesion molecules, whereas 2C11C additionally evokes phagocytosis of T-cells by macrophages.

### Absence of Anergy Induction in CD4^+^T-Cells by Anti-CD3

Several reports suggest that anti-CD3 mAbs induce T-cell anergy (13, 26). *Egr2* is one of the representative genes involved in anergy induction, and EGR-2 promotes the expression of downstream transcription factors such as CBL-B, GRAIL, ITCH, and DGKα, which ubiquitinate signal factors required for T-cell responses, keeping T-cells anergic (27). CD4^+^FOXP3^-^CD44^+^CD73^+^FR4^+^ polyclonal cells are considered to be anergic CD4^+^T-cell subsets in healthy hosts (28), and we confirmed that this population expressed EGR-2 intracellularly in the steady state (data not shown). We examined the mRNA expression of the indicated anergy-related genes in splenic CD4^+^T-cells by quantitative real-time PCR (**Figure S2A**), as well as the ratio of CD4^+^FOXP3^-^CD44^+^CD73^+^FR4^+^ cells to all CD4^+^T-cells in the spleen by flow cytometry (**Figures S2B, C**) after repeated antibody injections. These results suggest that neither 2C11S nor 2C11C induces anergy in CD4^+^T-cells, at least in our conditions.

### Suppression of Autoantibody Production in BWF1 Mice After 2C11S Treatment

**Figure 2:**
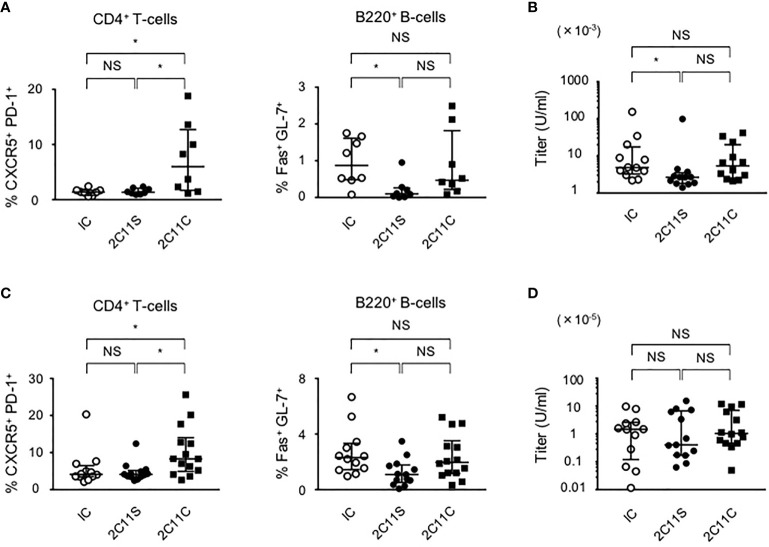
Suppression of autoantibody production after 2C11S treatment. **(A)** Splenic CD4^+^CXCR5^+^PD-1^+^ and B220^+^Fas^+^GL7^+^ cells were evaluated after each antibody treatment in the early phase. The ratio of Tfh cells to all CD4^+^T-cells and the ratio of GCB cells to all B220^+^B-cells were calculated. **(B)** Plasma anti-dsDNA IgG titers after the treatment were measured and displayed on a logarithmic scale. **(C)** Splenic Tfh and GCB cells after each antibody treatment in the late phase. The ratio of Tfh cells and GCB cells was calculated. **(D)** Plasma anti-dsDNA IgG titers after the treatment in the late phase were measured and displayed on a logarithmic scale (NS, Non Significance, *p < 0.05).

To investigate the short-term effect of anti-CD3 mAbs on lupus, each antibody was injected for 1 month to BWF1 mice at the age of 10 or 20 weeks reflecting early- and late-phase treatment, respectively. Splenic Tfh and GCB cells are known to be involved in high-affinity IgG antibody production (29, 30). In the early phase of treatment, 2C11S, but not 2C11C, reduced plasma anti-ds DNA IgG titers associated with the reduction of GCB cells but did not affect the differentiation of Tfh cells (**Figures 2A, B**). In the late-phase treatment, 2C11S did not reduce the IgG titers despite the reduction of GCB cells (**Figures 2C, D**). Collectively, the 2C11S-mediated reduction of GCB cells could affect the autoantibody production in the early phase of lupus.

### Unaffected Splenic Th1, Th17, or Treg Cells by Anti-CD3 Treatment

Previous reports indicate that anti-CD3 induces an expansion of Tregs that could contribute to the amelioration of autoimmune diseases (14, 31). To evaluate the differentiation of each effector CD4^+^T-cell subset, splenic CD4^+^IFN-γ^+^, CD4^+^IL-17a^+^, and CD4^+^CD25^+^FOXP3^+^ Tregs by anti-CD3 treatment in the late phase of lupus was stained and detected by flow cytometry. Combined with the data of splenic Tfh cell ratio (**Figures 2A, C**), anti-CD3 treatment does not influence the induction of Tregs or differentiation of effector T-cells (**Figures S4A**).

### 2C11S But Not 2C11C Attenuates Nephritis and Tissue Inflammation in BWF1 Mice

**Figure 3:**
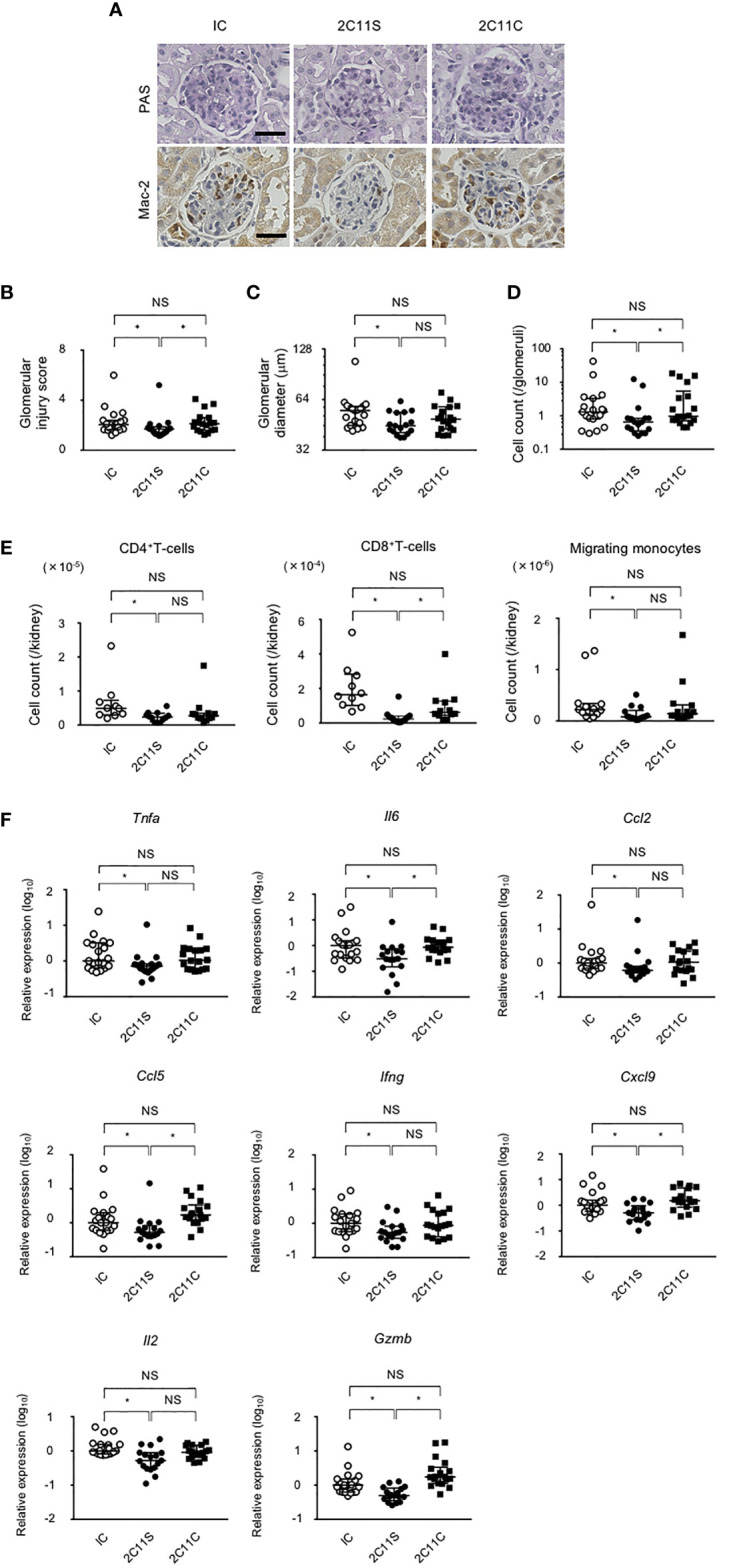
Attenuation of lupus nephritis and renal inflammation in 2C11S-treated mice. **(A)** PAS and Mac-2 immunostaining of renal glomeruli in each group. Original magnification, ×400. Scale bars, 50 μm. **(B)** The scoring for renal histopathological glomerular injury in each group. **(C)** The mean value of the maximum diameter of glomeruli in each group was displayed on a logarithmic scale. **(D)** The average count of Mac-2-positive migrated monocytes in glomeruli was displayed on a logarithmic scale. **(E)** The absolute number of each fraction of leukocytes in kidneys. **(F)** The relative mRNA expression of indicated cytokines and chemokines to *Gapdh* in the kidney was evaluated. Log10-fold differential expression is displayed (NS, Non Significance, *p < 0.05).

BWF1 mice develop glomerulonephritis with severe proteinuria starting at 5–6 months of age (32). After 1 month of weekly treatment with anti-CD3 in the late phase of lupus, kidney sections were evaluated and the histopathological glomerular injury was scored (**Figure 3A**). 2C11S significantly reduced the injury score, improved glomerular enlargement (**Figures 3B, C**), and significantly reduced the number of infiltrating monocytes detected by staining for the Mac-2 antigen (**Figures 3A, D**). CD4^+^T-cells, CD8^+^T-cells, CD11b^+^F4/80^high^ tissue-resident macrophages, and CD11b^+^F4/80^mid-low^ migrating monocytes were isolated from the kidneys and counted. 2C11S reduced significantly the number of CD4^+^T-cells, CD8^+^T-cells, and migrating monocytes (**Figure 3E**), but not that of tissue-resident macrophages (data not shown). Especially, gene expression levels of inflammatory cytokines such as TNFα and IL-6 and of chemokines inducing accumulation of monocytes were significantly reduced by 2C11S treatment in the whole kidneys (**Figure 3F**). The expression of *Il17a* was undetectable in all groups. These results showed that 2C11S, but not 2C11C, attenuates lupus nephritis and suppresses renal inflammation following a short-term treatment.

### Reduced Proteinuria and Prolonged Survival for the Long-Term Treatment of Anti-CD3

**Figure 4:**
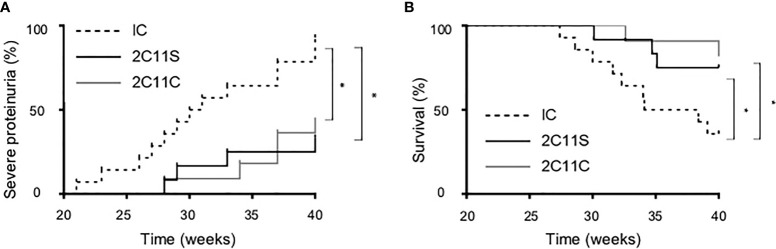
Reduced proteinuria and prolonged survival in the long-term treatment by both 2C11S and 2C11C. **(A)** The percentage of mice developing severe proteinuria (UP/Cr < 10 g/gCr) in each group was shown with the Kaplan–Mayer survival graph (*p < 0.05). **(B)** The survival of mice (weeks of age) in each group was shown with the Kaplan–Mayer survival graph (*p < 0.05).

To investigate the long-term effects of each antibody, the antibody treatment was continued until 40 weeks of age or until death while monitoring TCR density to be 50% or less by flow cytometry. Severe proteinuria was defined as uTP/uCr ratio (UP/Cr) >10 g/gCr. Both 2C11S and 2C11C reduced significantly proteinuria and prolonged survival (**Figures 4A, B**). The results indicate that 2C11S substantially exhibits therapeutic effects on lupus and nephritis, while treatment with 2C11C showed similar effects on ameliorating lupus, which could result from complicated mechanisms other than TCR reduction such as T-cell depletion and mitogenesis.

### Attenuated Autoimmunity in Lupus-Prone Heterozygous CD3ζ-Deficient Mice

**Figure 5:**
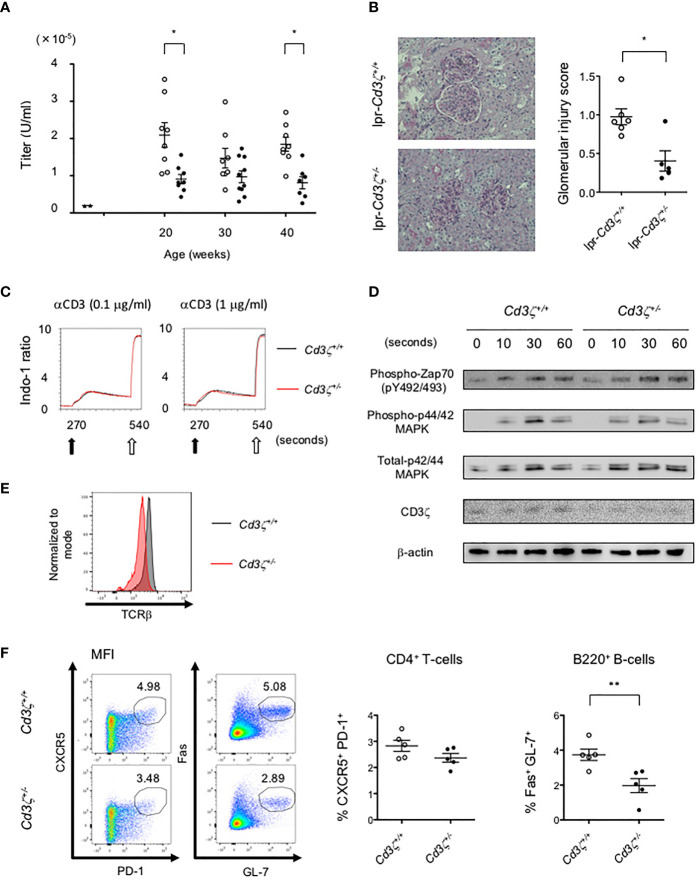
Suppression of the severity of lupus in B6lpr*-Cd3z^+/-^* mice, and reduced TCR expression with normal T-cell development and TCR signaling in B6*-Cd3z^+/-^* mice. **(A)** The titers of the plasma anti-dsDNA IgG antibody in each group; wild-type (black triangle), B6lpr*-Cd3z^+/+^* mice (white circle), and B6lpr*-Cd3z^+/-^* mice (black circle) mice were measured. **(B)** The PAS staining of the renal cortex was shown on the left. Original magnification, ×100. Histopathological glomerular injury score was shown on the right. **(C)** Intracellular Ca^2+^ influx after stimulation by 0.1/1 μg/ml of anti-CD3 antibody (black arrow) and 1 μg/ml of ionomycin (white arrow) was measured. **(D)** Western blot analysis in time series with CD4^+^T-cells after 1 μg/ml of anti-CD3ε antibody loading. Each band at the indicated time was shown. **(E)** TCRβ intensity on splenic CD4^+^T-cells at 6 weeks of age in each mouse. **(F)** Splenic CD4^+^CXCR5^+^PD-1^+^ and B220^+^Fas^+^GL-7^+^ cells after NP-CGG immunization were evaluated. Flow cytometric contour plots were shown on the left, and the ratio of these cells was shown on the right (*p < 0.05, **p < 0.01).

To investigate the effects of reduced TCR expression on the development of pathology in lupus, *Cd3z^+/-^* heterozygote mice were crossed into B6lpr mice (B6lpr-*Cd3z^+/-^* mice), which were found to express reduced amounts of TCR on the surface membrane of T-cells. The lupus nephritis-related pathology was attenuated in B6lpr-*Cd3z^+/-^* mice, along with suppressed autoantibody titers when compared with B6lpr-*Cd3z^+/+^* mice (**Figures 5A, B**). Although the expression of CD3ζ was decreased, B6-*Cd3z^+/-^* mice had a normal TCR signaling response following stimulation with a CD3 antibody (**Figures 5C, D**), T-cell development (**Figure S5A**), and TCR repertoire (**Figure S5B**), despite the fact that the intensity of surface TCR was reduced to 60% of that seen in *Cd3z^+/+^* mice (**Figure 5E**). Furthermore, when B6-*Cd3z^+/-^* mice were immunized with NP-CGG, the formation of GCB cells was significantly reduced compared to wild-type mice, despite the similar rates of differentiation of Tfh cells (**Figure 5F**). On the other hand, T-cell-independent antibody response by NP-Ficoll in B6-CD3ζ heterozygous mice was similar to that in wild-type mice (data not shown). These results indicate that TCR reduction does not influence T-cell development or signaling, but it affects effector T-cell function and the generation of GCB cells.

## Discussion

In this study, we have revealed the functional difference between 2C11C and 2C11S *in vitro*, *in vivo*, and in the lupus-prone mice. 2C11S was found to have sufficient potential to suppress the disease progression in lupus in both short- and long-term treatment. Mechanistically, 2C11S induced more sustained TCR reduction than 2C11C and induced lymphocyte trapping into the vessels *via* activated adhesion molecules leading to a temporal decrease in the number of peripheral T-cells. On the other hand, 2C11C induced T-cell expansion and cytokine production, as well as T-cell depletion through phagocytosis by macrophages, which were caused by the functional Fc region. Lastly, study of lupus-prone heterozygous CD3ζ-deficient mice indicated that the reduction of surface TCR density on T-cells is sufficient to attenuate lupus disease progression. Taken together, reduction of cell surface TCR, the predominant action of 2C11S, is effective for the treatment of lupus. It is expected that our results provide a better understanding about the precise mechanism of action of non-mitogenic anti-CD3.

In addition to TCR reduction, anti-CD3 is suggested to have other distinct effects on T-cells such as T-cell anergy, apoptosis, and Treg induction (12–14). However, the data of repeated injection of anti-CD3 have shown that 2C11C or 2C11S does not induce T-cell anergy (**Figures S2A, B**, **S3A, B**). Most studies on T-cell anergy resort to an operational definition (a state of tolerance induced by defective TCR stimulation) to classify T-cells as anergic, and distinctive markers for anergy across T-cell subsets and across species have yet to be clearly identified (27). Moreover, the functional properties of anergic T-cells remain controversial because anergic T-cells are reported to not only maintain the unresponsive state but also have additional functions: induction of peripheral Tregs, IL-10 production, and plasticity to adopt an activated phenotype (28, 33). Further experiments will be needed to clarify these unresolved problems about T-cell anergy.

A single injection of either 2C11C or 2C11S did not induce significant T-cell apoptosis *in vivo* (**Figure S1D**). Several reports suggest that anti-CD3 induces activation-induced- or programmed cell death of T-cells as a result of excessive downstream TCR signaling (12), which is supported as the primary immunosuppressive effect of anti-CD3 (31, 34). However, the observed T-cell apoptosis is partial and most of T-cells survive after antibody administration (31). In addition, a report that tracked the dynamics of effector T-cells *in vivo* using adaptive transfer of T-cells from antigen-specific TCR transgenic mice showed that anti-CD3 treatment did not deplete preferentially the effector T-cells and did not alter their proportion to total T-cells in target organs (35). Therefore, it is suggested that apoptosis is not a dominant immunosuppressive mechanism of anti-CD3.

Anti-CD3 is reported to promote conversion or expansion of CD4^+^CD25^+^FOXP3^+^ Tregs (14, 31) due to the increase of the ratio of CD4^+^CD25^+^FOXP3^+^ cells and the relative *Foxp3* gene expression in CD4^+^T-cells. However, other reports dispute this suggestion (35–38) as do our data presented in **Figure S4A**. The validation using FOXP3 reporter mice has shown that anti-CD3 does not induce additional FOXP3 expression in CD4^+^FOXP3^-^ T-cells, and selective CD4^+^FOXP3^-^ T-cell reduction increases the ratio of FOXP3^+^Tregs transiently but not the absolute number (35–37). To investigate whether the selective T-cell reduction results from the preferential T-cell trapping in the population, further experiments are necessary. These findings and our results show that the therapeutic effect of anti-CD3 does not contribute to the induction of Tregs.

It has been suggested that the rapid reduction of peripheral T-cell number after administration of anti-CD3 is mainly due to T-cell apoptosis (12, 39, 40). However, our validation using neutralizing antibodies to respective integrins indicates that 2C11S promotes T-cell trapping through the interaction between adhesion molecules on T-cells and vessels. The T-cell adhesion by anti-CD3 is thought to result from the conformational change of integrins (24) and the increase in their expression (41). Although we did not clarify whether T-cell trapping contributes to immunosuppression, T-cell number is mostly recovered 96 h after anti-CD3 injection (**Figure 1B**), suggesting that trapping is transient and has a lower impact on the T-cell responses. To clarify the precise impact on the immunomodulation, additional models and evaluation systems will be necessary.

In the short-term treatment of lupus, 2C11S did not affect splenic T-cell differentiation despite sustained TCR reduction (**Figure S4A**). The research on TCR signaling has introduced several models of reduced TCR density, and their validation has indicated that the major contributor of T-cell activation is TCR affinity for cognate antigens and that only a small number of high-affinity TCR is required to induce T-cell activation (42–44). In our study, the duration of short-term treatment was set for about 1 month, which might be sufficient for T-cells to undergo comparable activation and differentiation against autoantigens independently of TCR reduction in each treatment.

2C11S suppressed the differentiation of splenic GCB cells and reduced renal infiltration of migrated monocytes (**Figures 3A, C**, **4E**). The relationship between TCR avidity and T-cell responses was previously analyzed by modulating TCR density, suggesting that effector functions of T-cells are suppressed in response to the reduction of TCR number (43, 45, 46). Therefore, it is suggested that 2C11S-mediated TCR reduction attenuates the pathology of lupus *via* suppression of abnormal cell signaling caused by activated T-cells in each peripheral organ. This suggestion is also supported by the results obtained in TCR stimulation using external antigens: NP-CGG and concanavalin A (**Figures 2D–F**, **5F**).

Although high-affinity IgGs generally originate from the secondary follicle/germinal center, they are also suggested to occur in extrafollicular regions. Moreover, CXCR5^-^CXCR3^+^PD1^high^ extrafollicular T-cells, which help class switching by B cells in extrafollicular loci, were recently identified in SLE patients (47). These IL-10-producing extrafollicular T-cells are also reported to accumulate into the renal tubulointerstitial area in proliferative lupus nephritis and might contribute to the renal pathogenesis. We could not find a significant difference of renal IL-10 expression among controls and treatment groups (data not shown); however, the possibility that reduction of TCR by 2C11S could affect the extrafollicular T-cell response cannot be ruled out.

In the late-phase treatment of lupus, 2C11S did not significantly suppress anti-dsDNA antibody production in the late phase (**Figure 3D**). Anti-dsDNA antibody titers increased in all control samples in the early phase, whereas some of them spontaneously decreased in the late phase despite the advanced differentiation of GCB cells (data not shown). These results suggest that deposition of affinity-matured autoantibodies in peripheral tissues was faster than their production and counteracted the effect of reduced autoantibody production by 2C11S.

In our long-term treatment studies, 2C11C as well as 2C11S reduced proteinuria and prolonged survival. In this protocol, the doses of 2C11S and 2C11C were adjusted to reduce TCR density by 50% or less in peripheral blood lymphocytes. Both 2C11S and 2C11C could increase the risk of infectious complications by suppressing T-cell function; however, 2C11S seems to be more advantageous in terms of safety than 2C11C because 2C11C, but not 2C11S, could induce lymphopenia by depleting T-cells (**Figures 1G, H**). Adverse effects of anti-CD3 should be still considered carefully before clinical use.

In conclusion, we have demonstrated that non-mitogenic anti-CD3 antibody 2C11S has therapeutic potential for lupus by selectively targeting the TCR/CD3 complex without causing T-cell death or stimulation.

## Data Availability Statement

The original contributions presented in the study are included in the article/**Supplementary Material**. Further inquiries can be directed to the corresponding author.

## Ethics Statement

The animal study was reviewed and approved by the Animal Experiment Committee of The Institute of Medical Science, Osaka University, and the Beth Israel Deaconess Medical Center Institutional Animal Care and Use Committee (IACUC).

## Author Contributions

All authors were involved in drafting the article and revising it critically for the intellectual content, and all authors approved the final version to be published. GT had full access to all data in this study and take responsibility for the integrity of the data.

## Funding

This work was supported by JSPS KAKENHI Grant Numbers 15H06374 (MMi), 17K10001 (MMi), and 20K16131 (MMo) and PHS RO1AI42269 (GCT). The authors do not have any other financial support which could create a potential conflict of interest or the appearance of a conflict of interest concerning the work in the manuscript.

## Conflict of Interest

The authors declare that the research was conducted in the absence of any commercial or financial relationships that could be construed as a potential conflict of interest.

## Publisher’s Note

All claims expressed in this article are solely those of the authors and do not necessarily represent those of their affiliated organizations, or those of the publisher, the editors and the reviewers. Any product that may be evaluated in this article, or claim that may be made by its manufacturer, is not guaranteed or endorsed by the publisher.
