# Supplementary material for: Reduction of Cell Surface T-Cell Receptor by Non-Mitogenic CD3 Antibody to Mitigate Murine Lupus
Source: Front Immunol. 2022 Mar 28;13:855812. doi: 10.3389/fimmu.2022.855812 (PMC8995471; doi:10.3389/fimmu.2022.855812)
Supplement: Supplementary file 1 [file DataSheet_1.docx]

**
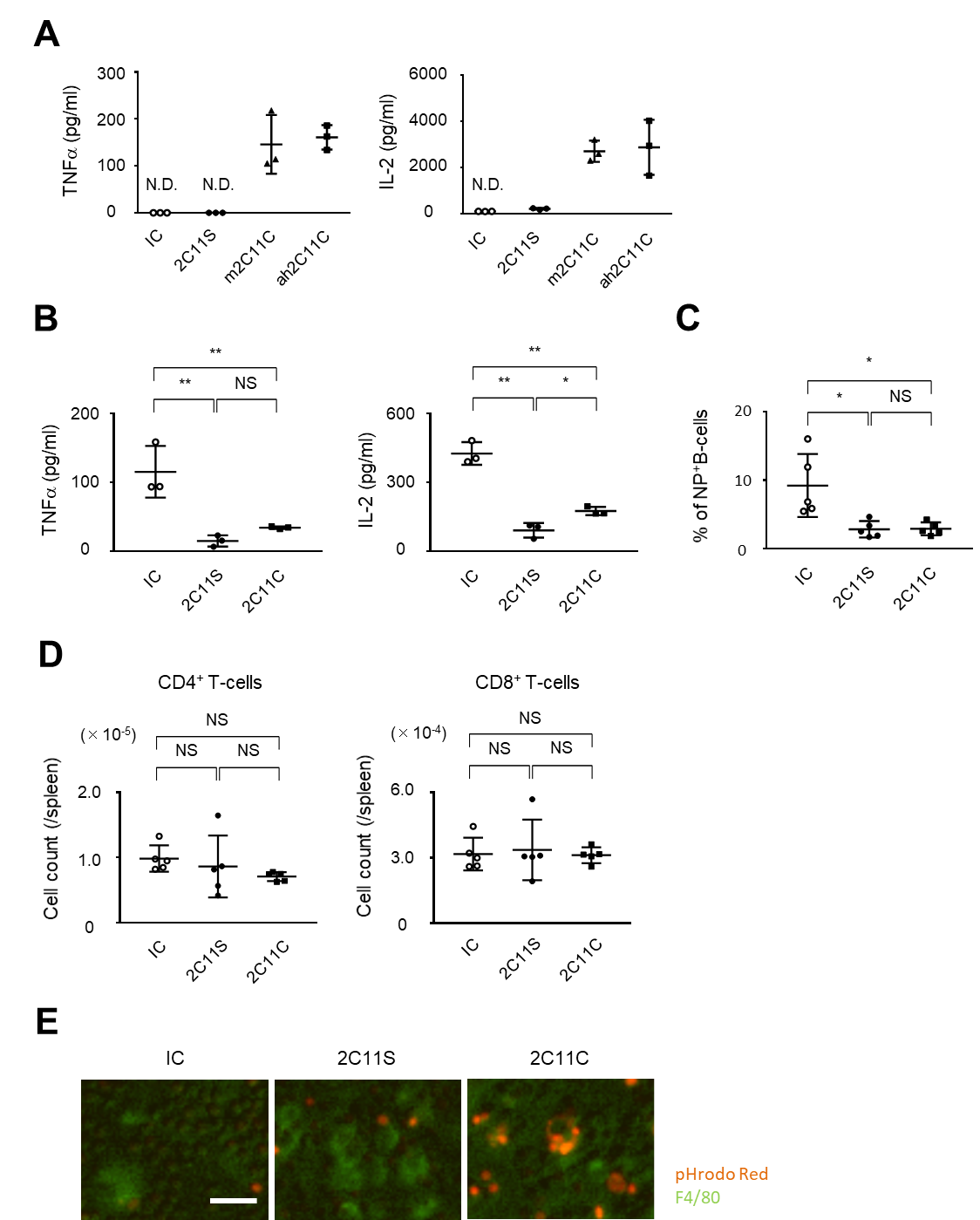
**

**Supplementary Figure 1. Functional difference between 2C11C and 2C11S.** (A) Levels of plasma TNF-α in 1 hour and IL-2 in 2 hours after injection of each antibody were measured. (B) Splenic pan T-cells harvested 96 hours after injection of each antibody were incubated with 2 μg/ml concanavalin A for 24 hours. Levels of TNF-α and IL-2 in the supernatants were measured. (C) NP-specific B-cells in NP-CGG immunized mice after each antibody treatment were shown. (D) Apoptotic CD4^+^T-cells and CD8^+^T-cells in B6 mice 2 hours after injection of each antibody were detected and counted (N.D.; not detected, *P < 0.05, **P < 0.01). (E) The representative image at 2 hours was shown in each group. Original magnification, ×400. Scale bars, 25 μm.


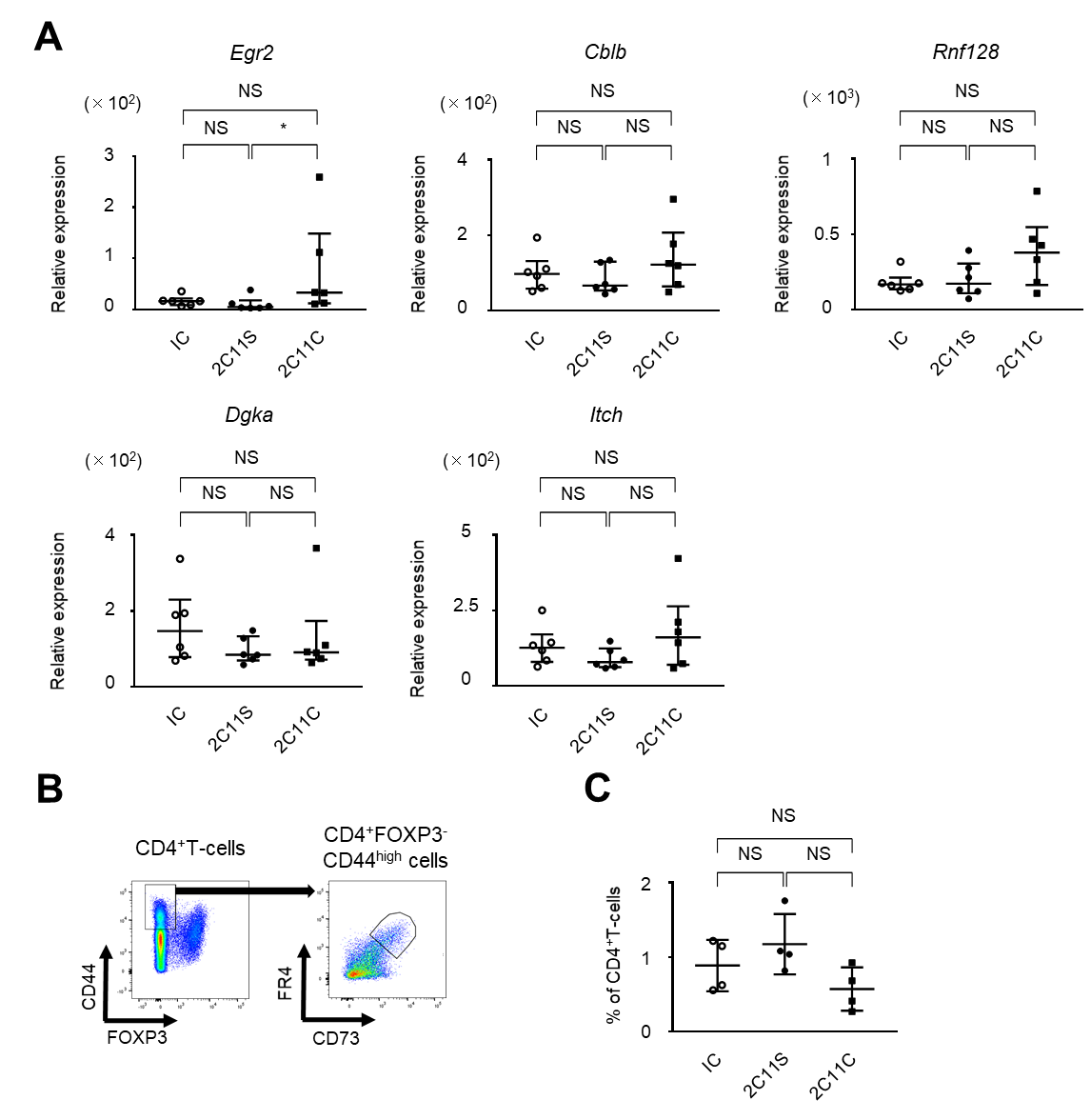


**Supplementary Figure 2. No significant induction of anergy in CD4^+^T-cells of B6 mice treated by anti-CD3.** (A) The relative mRNA expression of indicated genes to *Gapdh* in splenic CD4^+^T-cells of B6 mice after repeated injection of each antibody was examined. (B) Flow cytometric gating strategy for detection of anergic CD4^+^T-cells. (C) Splenic CD4^+^FOXP3^-^CD44^high^CD73^high^FR4^high^ cells were detected and the ratio of these cells to all splenic CD4^+^T-cells was calculated (*P < 0.05).


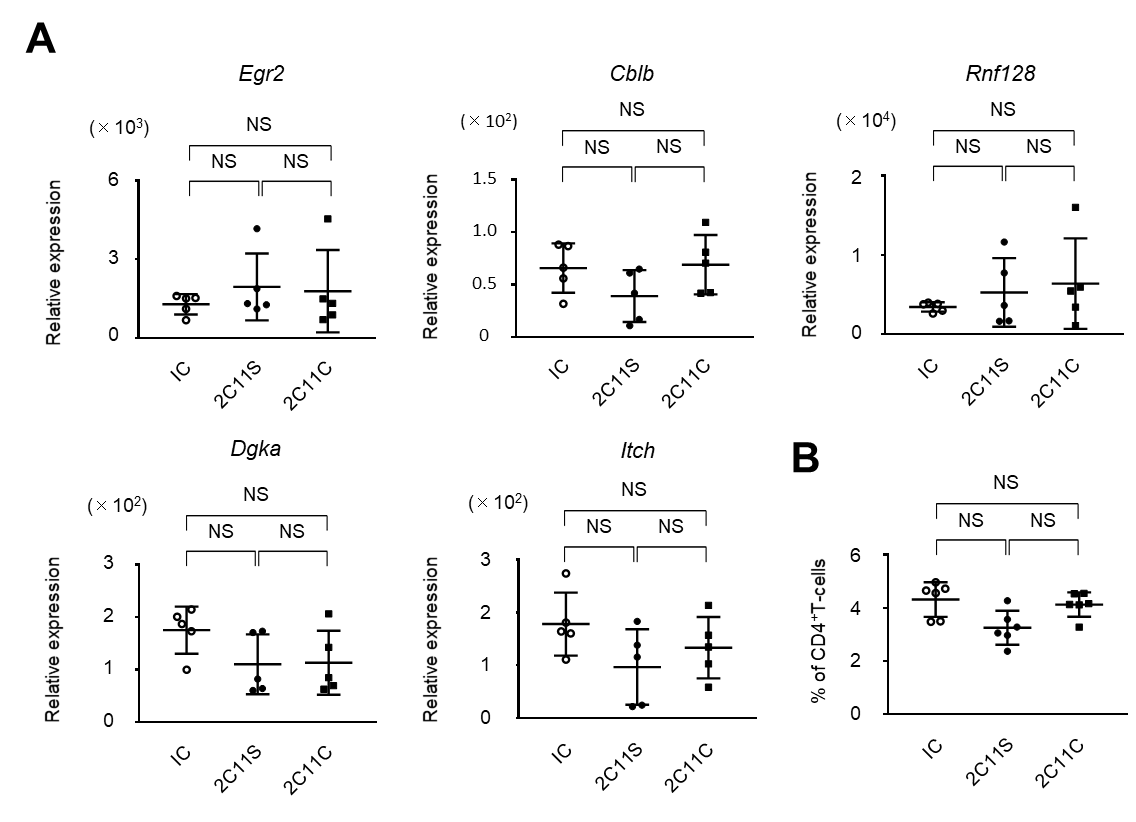


**Supplementary Figure 3. No significant induction of anergy in CD4^+^T-cells of BWF1 mice after anti-CD3 treatment.** (A) The relative mRNA expression of indicated genes to *Gapdh* in splenic CD4^+^T-cells of BWF1 mice after repeated injection of each antibody was examined. (B) Splenic CD4^+^FOXP3^-^CD44^high^CD73^high^FR4^high^ cells were detected and the ratio of these cells to all splenic CD4^+^T-cells was calculated (*P < 0.05).

**
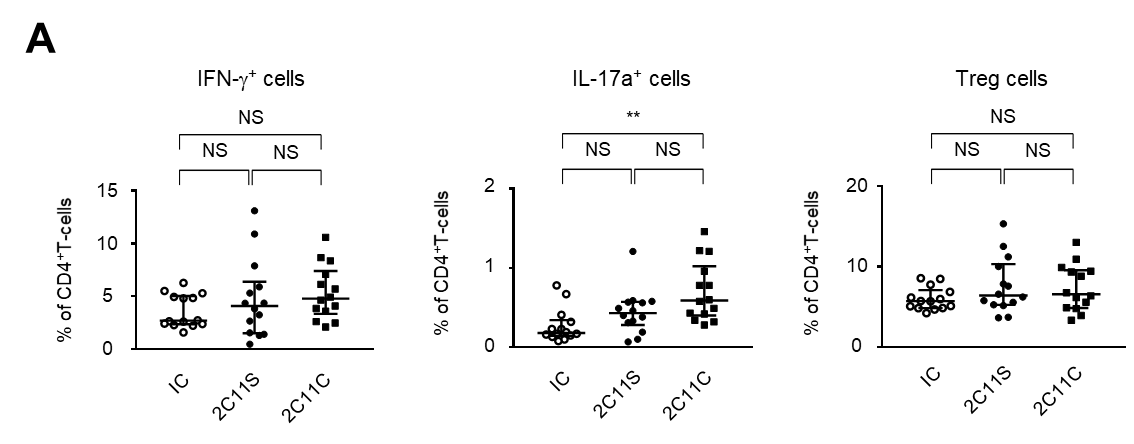
**

**Supplementary Figure 4. The differentiation of splenic Th1, Th17, and Treg cells after anti-CD3 administration.** (A) After the short-term treatment in the late phase, the ratio of CD4^+^IFN-γ^+^, CD4^+^IL-17a^+^, and CD4^+^CD25^+^FOXP3^+^ cells to all CD4^+^T-cells in spleen was evaluated (*P < 0.05, **P < 0.01).

**
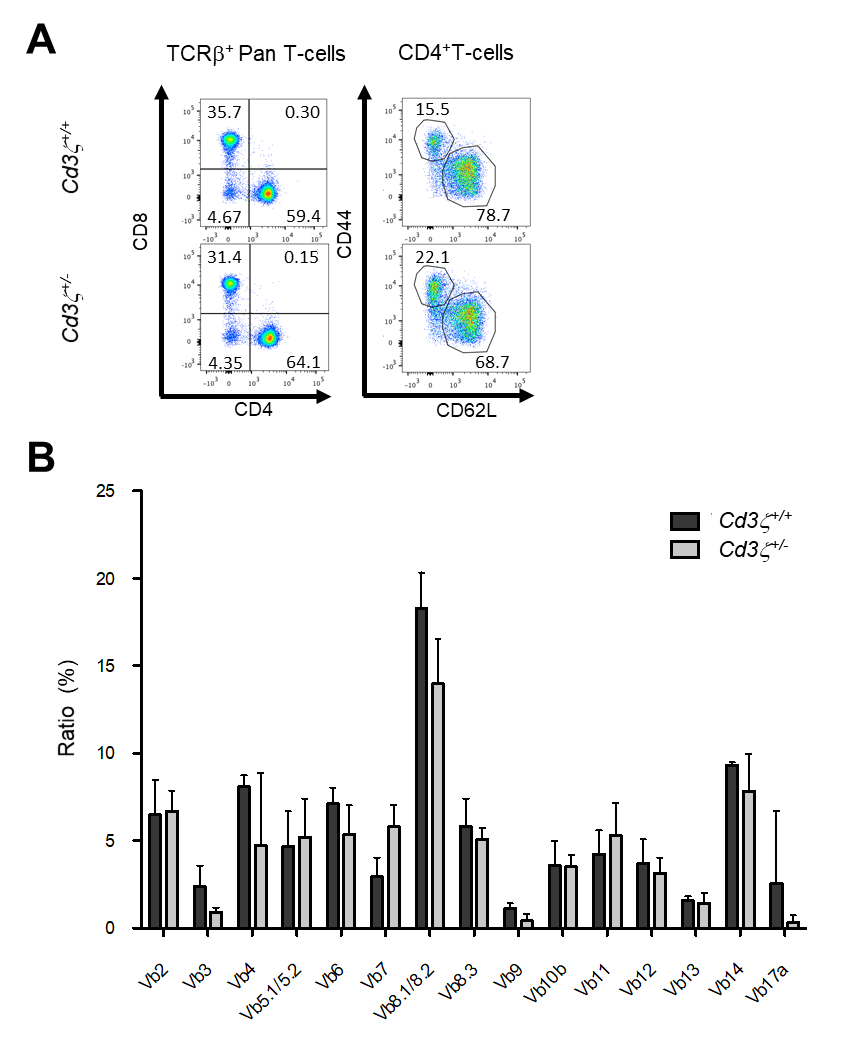
**

**Supplementary Figure 5. The properties of T-cells in B6-*Cd3z^+/-^* mice.** (A) Flow cytometric gating strategy for detection of T-cell subpopulation. Gates of viable CD3^+^ Pan T-cells were shown on the left and gates of CD3^+^CD4^+^ T-cells were shown on the right. (B) Quantitative analyses of TCR-Vβ families on thymic CD4 single-positive cells in each group were performed. The ratio of each Vβ subclass was calculated (*P < 0.05).

**Supplementary Table 1. Primer sequences used for quantitative real-time PCR.**

| Genes | Forward, 5’→3’ | Reverse, 5’→3’ |
| --- | --- | --- |
| *Ccl2* | catccacgtgttggctca | gatcatcttgctggtgaatgagt |
| *Ccl5* | tgcagaggactctgagacagc | gagtggtgtccgagccata |
| *Cxcl9* | cttttcctcttgggcatcat | gcatcgtgcattccttatca |
| *Il6* | gctaccaaactggatataatcagga | ccaggtagctatggtactccagaa |
| *Ifng* | atctggaggaactggcaaaa | ttcaagacttcaaagagtctgagg |
| *Tnfa* | tgaggaaggctgtgcattg | ggccttcctaccttcagacc |
| *Il2* | gctgttgatggacctacagga | Ttcaattctgtggcctgctt |
| *Gzmb* | gctgctcactgtgaaggaagt | tggggaatgcattttaccat |
| *Egr2* | ctacccggtggaagacctc | aatgttgatcatgccatctcc |
| *Cblb* | acaggctggcgagtgttc | Gagcctggcgatgtgact |
| *Rnf128* | gcatcatctggatatgcttcag | ggtttaccagctgtagattttcatt |
| *Dgka* | ctgggcactggaaatgatct | aatctttctcaaattctcaccttca |
| *Itch* | caagctttctttgagggcttta | gcatcccacacagaagaacc |
| *Gapdh* | aactttggcattgtggaagg | acacattgggggtaggaaca |

Abbreviations: *Ccl2*, chemokine C-C motif ligand 2; *Ccl5*, chemokine C-C motif ligand 5; *Cxcl9*, chemokine C-X-C motif ligand 9; *Il6*, interleukin 6; *Ifng*, interferon-gamma; *Tnfa*, tumor necrosis factor alfa; *Il2*, interleukin 2; Gzmb, granzyme B; *Egr2*, early growth response 2; *Cblb*, Casitas B-lineage lymphoma b; *Rnf128*, ring finger protein 128; *Dgka*, diacylglycerol kinase, alpha; *Itch*, itchy, E3 ubiquitin-protein ligase; *Gapdh*, glyceraldehyde-3-phosphate dehydrogenase. The primers’ sequences were designed according to the sequence of each gene deposited in the GenBank database.
